# Age-related variation in the association between intraoperative dynamic cerebral autoregulation impairment proportion and postoperative neutrophil-to-lymphocyte ratio: an exploratory observational study

**DOI:** 10.3389/fnagi.2026.1898034

**Published:** 2026-09-01

**Authors:** Chengying Ji, Bokang Yang, Jiayi Xie, Jinxiang Xie, Xiaodong Su, Yatao Liu

**Affiliations:** 1 The First School of Clinical Medicine, Lanzhou University, Lanzhou, Gansu, China; 2 Department of Anesthesiology and Surgery, First Hospital of Lanzhou University, Lanzhou, Gansu, China

**Keywords:** cerebral oximetry index, dynamic cerebral autoregulation, inflammation, neutrophil-to-lymphocyte ratio, postoperative

## Abstract

**Background:**

Dynamic cerebral autoregulation (dCA) impairment during surgery has been associated with adverse neurologic outcomes. Whether the burden of impaired dCA is related to the postoperative peripheral inflammatory response, and whether this association varies with age, remains uncertain.

**Objective:**

To examine whether age modifies the association between intraoperative dCA impairment proportion and the neutrophil-to-lymphocyte ratio on postoperative day 3 (NLR3).

**Methods:**

This single-center exploratory observational study included 57 adults undergoing elective major abdominal surgery. dCA impairment proportion was calculated as cumulative impaired dCA duration divided by valid dCA monitoring duration; impaired dCA was defined as a cerebral oximetry index (COx) ≥ 0.3 in at least one hemisphere. NLR3 was modeled using a Gamma generalized linear model with a log link. The model included continuous age, dCA impairment proportion, their interaction, baseline neutrophil-to-lymphocyte ratio (NLR0), operation duration, and sex. Robust variance estimation, bootstrap resampling, leave-one-out analysis, and alternative outcome and exposure definitions were used to assess model stability.

**Results:**

The age × dCA impairment proportion interaction was positive (Exp(B) = 1.193 per 10-year × 10-percentage-point increment; model-based 95% confidence interval [CI], 1.051–1.354; *P* = 0.006). The point estimate was unchanged with heteroskedasticity-consistent type 3 (HC3) robust standard errors, although the CI widened (95% CI, 0.975–1.459; *P* = 0.086). The 2,000-replicate bootstrap percentile interval for the interaction coefficient was 0.051–0.370. A log-transformed NLR3 sensitivity model yielded a similar interaction (HC3 *P* = 0.040), whereas no interaction was observed when cumulative impaired dCA duration was used (HC3 *P* = 0.915). The age-conditional slopes were imprecise. Longer operation duration was associated with higher NLR3 (Exp(B) = 1.214 per hour; 95% CI, 1.041–1.414; *P* = 0.013).

**Conclusion:**

In this cohort, the modeled association between dCA impairment proportion and NLR3 became more positive with increasing age. The consistency of the interaction depended on the method used to estimate uncertainty and on whether dCA impairment was expressed as a proportion or as cumulative impaired dCA duration. These findings identify an age-related pattern that merits prospective validation using standardized dCA monitoring and serial inflammatory measurements.

## 1 Introduction

Dynamic cerebral autoregulation (dCA) enables the cerebral circulation to buffer changes in systemic arterial pressure and maintain relatively stable cerebral perfusion (Tarumi and Zhang, 2023; Vu et al., 2024). During anesthesia and major surgery, blood pressure variation, anesthetic exposure, surgical stress, and changes in carbon dioxide tension may challenge this regulatory capacity (Brown et al., 2019). Sustained pressure-passive cerebral circulation can expose the brain to hypoperfusion or hyperperfusion and has been associated with postoperative delirium and other adverse neurologic outcomes (Zheng et al., 2015; Chuan et al., 2019).

The cerebral oximetry index (COx) provides a continuous bedside estimate of dCA by calculating the moving correlation between regional cerebral oxygen saturation (rScO_2_), measured by near-infrared spectroscopy (NIRS), and arterial blood pressure. When rScO_2_ varies in parallel with arterial pressure, COx becomes more positive, indicating reduced buffering of pressure fluctuations (Brown et al., 2019; Tarumi and Zhang, 2023; Vu et al., 2024). A COx threshold of 0.3 has commonly been used to identify impaired autoregulation (Zhang et al., 2021).

Major surgery also triggers a systemic stress response involving neuroendocrine activation, leukocyte redistribution, tissue injury, and inflammatory signaling (Dobson, 2015; Pan et al., 2025). The neutrophil-to-lymphocyte ratio (NLR), obtained from routine blood counts, integrates two components of this response and has been used for perioperative risk assessment (Silva et al., 2024; Zhu et al., 2023). Because NLR is influenced by both neutrophil and lymphocyte counts and by multiple perioperative factors, it is best interpreted as a pragmatic marker of the postoperative peripheral response rather than a marker of a specific inflammatory pathway.

Most perioperative dCA studies have focused on neurologic outcomes, and the relationship between intraoperative dCA impairment burden and postoperative peripheral inflammatory markers has received less attention. Cumulative impaired dCA duration quantifies the absolute time spent above the COx threshold, whereas dCA impairment proportion describes the fraction of the valid dCA monitoring period spent with impairment. Evaluating both measures may help distinguish an association with proportional pressure passivity from an association that simply reflects longer monitoring or longer surgery.

Age is a biologically plausible source of heterogeneity because aging is accompanied by changes in vascular compliance, endothelial function (Burkhart et al., 2011; Ungvari et al., 2025), cerebrovascular reserve, autonomic regulation (Shen et al., 2026), and immune responsiveness (Bowirrat, 2022; Dilger and Johnson, 2008; Jurgens and Johnson, 2012; Niraula et al., 2012; Sun et al., 2024). We therefore hypothesized that the association between intraoperative dCA impairment burden and postoperative day-3 NLR (NLR3) would vary across the adult age range. The primary aim was to estimate the interaction between continuous age and dCA impairment proportion after adjustment for baseline NLR, operation duration, and sex. Cumulative impaired dCA duration and the conventional 65-years age grouping were evaluated as complementary analyses.

## 2 Materials and methods

### 2.1 Study design

This single-center exploratory observational study was nested within an ongoing registered cohort (ChiCTR2400089276) at the First Hospital of Lanzhou University. Participants were recruited between October 2024 and October 2025 and underwent elective major abdominal surgery under general anesthesia with continuous intraoperative dCA monitoring. Clinical care was not altered for the present analysis, and follow-up continued through postoperative day 3.

### 2.2 Participants

This study included all 61 patients from the parent cohort who underwent intraoperative dCA monitoring between October 2024 and October 2025. Adults undergoing elective major abdominal surgery were eligible when perioperative clinical data, continuous dCA monitoring, and postoperative day-3 blood counts were available. Exclusion criteria were severe preoperative organ dysfunction, a history of central nervous system disease, incomplete dCA monitoring, or missing NLR3. No additional selection was based on exposure or outcome. All patients exhibited at least some periods with COx ≥ 0.3; four were excluded because of incomplete monitoring data, leaving 57 patients for analysis.

### 2.3 Variables and outcomes

The primary outcome was NLR3, obtained from the postoperative day-3 blood count. Baseline NLR (NLR0) was obtained from the most recent preoperative blood count. The source variables NEUT0, LYMPH0, NEUT3, and LYMPH3 are reported in Table 1 using the labels retained from the clinical dataset.

**TABLE 1:** Characteristics of the study population.

Baseline characteristics	Total (*n* = 57)	Age < 65 (*n* = 31)	Age ≥ 65 (*n* = 26)
Sex, n (%)			
Male	42 (74%)	22 (71%)	20 (77%)
Female	15 (26%)	9 (29%)	6 (23%)
Age, years	62.5 ± 10.7	54.6 ± 7.9	71.8 ± 4.0
BMI, kg/m^2^	22.0 (21.2, 24.2)	22.0 (21.2, 24.0)	24.6 (22.2, 25.6)
ASA physical status, n (%)			
II	18 (32%)	13 (42%)	5 (19%)
III	39 (68%)	18 (58%)	21 (81%)
Surgical site, n (%)			
Pancreas	3 (5%)	1 (3%)	2 (8%)
Liver	7 (12%)	4 (13%)	3 (12%)
Stomach	30 (53%)	14 (45%)	16 (61%)
Intestine	17 (30%)	12 (39%)	5 (19%)
NEUT0	3.2 (2.5, 4.6)	3.2 (2.6, 4.5)	3.2 (2.4, 4.7)
LYMPH0	1.2 (1.0, 1.6)	1.4 (1.1, 1.6)	1.2 (0.9, 1.6)
NLR0	2.6 (1.8, 3.6)	2.5 (1.7, 3.6)	2.6 (1.8, 3.3)
NEUT3	6.6 (4.9, 8.1)	5.8 (4.2, 7.7)	6.9 (5.5, 8.3)
LYMPH3	0.9 (0.6, 1.1)	0.9 (0.6, 1.1)	0.9 (0.7, 1.0)
NLR3	8.0 (5.4, 10.2)	6.8 (4.9, 10.0)	8.0 (5.8, 10.5)
Operation duration, min	220.0 (180.0, 257.5)	220.0 (180.0, 240.0)	225.0 (180.0, 300.0)
Anesthesia duration, min	243.0 (202.5, 270.0)	243.0 (210.0, 270.0)	238.0 (199.8, 296.3)
Valid dCA monitoring duration, s	12220.0 (10055.0, 13840.0)	11990.0 (9340.0, 12980.0)	12710.0 (10067.5, 16445.0)
Cumulative impaired dCA duration, s	6270.0 (4705.0, 7755.0)	5600.0 (4490.0, 6700.0)	7460.0 (5492.5, 8662.5)
dCA impairment proportion	0.51 ± 0.11	0.49 ± 0.11	0.54 ± 0.10
Intraoperative medication			
Propofol, mg	1070.3 ± 318.2	1052.9 ± 297.5	1091.0 ± 346.1
Sufentanil, μg	36.0 (30.0, 42.5)	40.0 (30.0, 45.0)	35.0 (30.0, 40.0)
Remifentanil, μg	3300.0 (2940.9, 4067.5)	3360.0 (2975.0, 4035.0)	3279.2 (2420.0, 4125.0)
Norepinephrine, μg	937.5 (497.0, 1524.1)	826.7 (504.0, 1437.6)	1126.7 (467.5, 1939.3)
Blood loss, mL	200.0 (100.0, 300.0)	200.0 (100.0, 230.0)	200.0 (87.5, 300.0)
Urine output, mL	500 (325, 650)	500 (350, 700)	500 (300, 625)
Postoperative delirium, n (%)			
Yes	14 (25%)	7 (23%)	7 (27%)
No	43 (75%)	24 (77%)	19 (73%)

Data are presented as mean ± standard deviation (SD), median (interquartile range), or *n* (%). BMI, body mass index; ASA, American Society of Anesthesiologists; NEUT0/NEUT3 and LYMPH0/LYMPH3, source-variable labels for absolute neutrophil and lymphocyte counts (×10^9^/L), respectively, with numeric suffixes retained from the clinical dataset; NLR0, baseline neutrophil-to-lymphocyte ratio; NLR3, neutrophil-to-lymphocyte ratio on postoperative day 3; COx, cerebral oximetry index; dCA, dynamic cerebral autoregulation; n, number of participants. Impaired dCA was defined as COx ≥ 0.3 in at least one hemisphere; concurrent bilateral impairment was counted once. Valid dCA monitoring duration excluded periods of sensor detachment, arterial-line flushing, or evident signal loss. dCA impairment proportion was calculated as cumulative impaired dCA duration divided by valid dCA monitoring duration.

The primary exposure was dCA impairment proportion, calculated as cumulative seconds with COx ≥ 0.3 in at least one cerebral hemisphere divided by the total valid dCA monitoring duration. Cumulative impaired dCA duration was analyzed separately and was expressed per 10 min. Age was analyzed as a continuous variable in the primary model. Age categories of <65 and ≥65 years were used for descriptive presentation and a secondary interaction analysis.

The primary adjustment variables were NLR0, operation duration, and sex. Baseline NLR accounts for preoperative variation in the inflammatory marker, operation duration represents the duration and complexity of the surgical insult and is also related to the available monitoring interval, and sex was included as a biological covariate. Surgical site was described in Table 1 but was not entered as multiple sparse category indicators in the primary model. Postoperative delirium was summarized descriptively because it occurred after the intraoperative exposure.

### 2.4 Anesthesia and intraoperative management

All patients received standardized general anesthesia. Anesthesia was induced with intravenous propofol (1.5–2.5 mg/kg), sufentanil (0.3–0.5 μg/kg), and rocuronium (0.6–0.9 mg/kg) to facilitate tracheal intubation. After intubation, mechanical ventilation was initiated with a tidal volume of 6–8 mL/kg of predicted body weight, a respiratory rate of 12–16 breaths/min, and FiO2 of 0.5–0.6, adjusted to maintain end-tidal CO_2_ between 35 and 45 mmHg. Anesthesia was maintained with continuous infusion of propofol (4–6 mg⋅kg^−1^⋅h^−1^) and remifentanil (0.1–0.3 μg⋅kg^−1^⋅min^−1^), targeting a bispectral index of 40–60. Intermittent boluses of rocuronium were administered as needed to maintain neuromuscular blockade.

Intraoperative monitoring included invasive arterial blood pressure (via radial artery catheter), heart rate, pulse oximetry, electrocardiography, end-tidal CO2, nasopharyngeal temperature, and NIRS for rScO_2_. MAP was maintained within ±20% of baseline values; hypotension (MAP < 65 mmHg) was treated with intravenous norepinephrine to maintain blood pressure, and crystalloids (3–5 mL/kg) were administered if needed. Bradycardia (heart rate < 50 bpm) was treated with atropine (0.3–0.5 mg). Fluid management consisted of maintenance infusion of Ringer’s acetate solution at 4–6 mL⋅kg^−1^⋅h^−1^, with additional boluses for blood loss or hypotension. Blood transfusion was considered when hemoglobin dropped below 70 g/L or based on clinical judgment.

Before the end of surgery, a patient-controlled intravenous analgesia pump was connected, prepared with 150 μg sufentanil and 200 mg flurbiprofen axetil diluted with normal saline to a total volume of 150 mL, with a background infusion rate of 2 mL/h. Residual neuromuscular blockade was reversed with neostigmine (0.04 mg/kg) combined with atropine (0.02 mg/kg). Patients were extubated after meeting standardized criteria (awake, sustained head lift for >5 s, adequate tidal volume, and stable hemodynamics). All patients were transferred to the post-anesthesia care unit for close monitoring for 1 h before returning to the general ward.

### 2.5 dCA monitoring and exposure construction

Bilateral NIRS sensors were placed on each patient’s forehead to continuously monitor left- and right-sided rScO_2_. We monitored rScO_2_ using a Mindray BeneVision N12 patient monitor with its rScO_2_ module (Shenzhen Mindray Bio-Medical Electronics Co., Ltd.). The probe operated at a raw sampling frequency of 100 Hz, and the system automatically calculated and recorded rSO2 values at 2-s intervals. Arterial blood pressure (ABP) was continuously measured via an invasive arterial catheter connected to the same monitor, and its sampling frequency was matched to that of the rScO_2_ recording. Invasive MAP and bilateral rScO_2_ signals were synchronously transferred to ICM+ software (Cambridge Enterprise, University of Cambridge, UK)^1^. MAP and rScO_2_ signals were averaged over 10-s intervals, and Pearson correlation coefficients were calculated using a 300-s moving window comprising 30 consecutive data points, thereby generating separate left- and right-sided COx values for bilateral dCA assessment. Impaired dCA was defined as a COx value ≥ 0.3 in at least one cerebral hemisphere. Monitoring commenced once stable, valid signals were obtained after anesthesia induction and continued until nonessential physiological monitors were removed shortly before the end of surgery. Data recorded during sensor detachment, arterial-line flushing, or evident signal loss were excluded. Vasoactive drug administration and blood pressure fluctuations were retained as part of the actual intraoperative physiological process and were not used to redefine the COx threshold.

### 2.6 Data sources and study size

Demographic, surgical, medication, fluid, blood-loss, delirium, and laboratory data were obtained from electronic medical records and the prospective cohort database. dCA variables were exported from ICM+. Postoperative delirium was assessed twice daily during the first three postoperative days using the Chinese version of the 3-Min Diagnostic Interview for Confusion Assessment Method (CAM)-defined Delirium (3D-CAM). Because the study used all eligible complete cases available from this nested cohort and was exploratory, no formal prospective sample-size calculation was performed.

### 2.7 Statistical analysis

Continuous variables are presented as mean ± standard deviation or median (interquartile range), according to their distribution; categorical variables are presented as counts and percentages. The primary analysis evaluated the interaction between continuous age and dCA impairment proportion in a unified regression model. One primary interaction was specified for interpretation, whereas conditional effects, alternative model forms, the 65-years grouping, and the cumulative impaired dCA duration exposure were treated as supportive analyses.

Neutrophil-to-lymphocyte ratio on postoperative day 3 was analyzed using a generalized linear model with a Gamma distribution and log link. Age was centered at the sample mean and scaled per 10 years. dCA impairment proportion was centered at its sample mean and scaled per 10 percentage points. Operation duration was expressed in hours. The model included age, dCA impairment proportion, their product term, NLR0, operation duration, and sex. Exponentiated coefficients, Exp(B), represent multiplicative changes in the expected NLR3.

The Gamma dispersion parameter was estimated by maximum likelihood. Model-based confidence intervals (CIs) and CIs based on heteroskedasticity-consistent type 3 (HC3) robust standard errors were reported because small samples can yield different uncertainty estimates. Model stability was further assessed with 2,000 nonparametric bootstrap resamples and leave-one-out refitting. Sensitivity analyses used log-transformed NLR3 in a linear model, log-transformed NLR0, an age-group × dCA impairment proportion interaction with age dichotomized at 65 years, and cumulative impaired dCA duration instead of dCA impairment proportion.

Collinearity was assessed using variance inflation factors (VIFs), tolerance, condition indices, and variance-decomposition proportions. Model convergence, dispersion, deviance, Pearson residuals, leverage, standardized residuals, and Cook’s distance were examined. All tests were two-sided. The Statistical Package for the Social Sciences (SPSS), version 26.0, was used for descriptive data verification, and R version 4.3.1 was used for regression modeling, diagnostics, bootstrap analysis, and sensitivity analyses.

## 3 Results

### 3.1 Participant flow and characteristics

**FIGURE 1:**
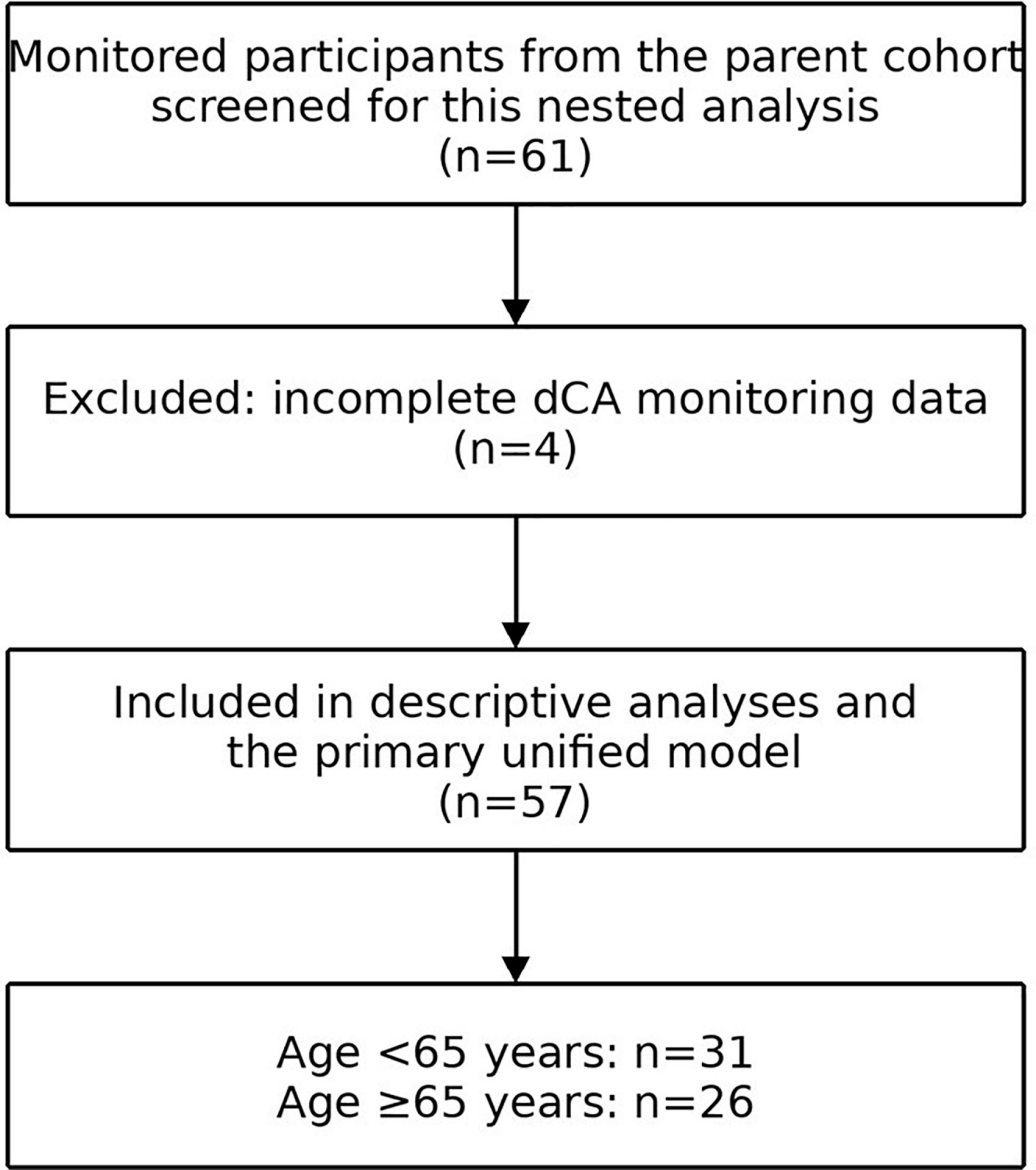
Participant flow for the nested study.

Of 61 monitored participants assessed for this nested study, four were excluded because dCA monitoring data were incomplete, and 57 were included in the analyses (Figure 1). Thirty-one participants were aged < 65 years and 26 were aged ≥ 65 years. Postoperative delirium occurred in 14 participants (24.6%). Patient and perioperative characteristics are summarized in Table 1.

Median NLR3 was 8.0 (interquartile range 5.4–10.2), with observed values ranging from 0.22 to 41.27. The distribution of NLR3 is shown in Supplementary Figures 1–4. Median valid dCA monitoring duration was 12,220 s, median cumulative impaired dCA duration was 6,270 s, and mean dCA impairment proportion was 0.51 ± 0.11.

### 3.2 Primary age × dCA impairment proportion interaction

In the Gamma-log model, the age × dCA impairment proportion coefficient was *B* = 0.176 (Exp(B) = 1.193; model-based 95% CI, 1.051–1.354; *P* = 0.006). With HC3 robust standard errors, the point estimate was unchanged, and the 95% CI was 0.975–1.459 (*P* = 0.086) (Table 2). The fitted interaction therefore indicated a progressively more positive association between dCA impairment proportion and expected NLR3 at older ages, with greater uncertainty under the robust variance estimator.

**TABLE 2:** Primary Gamma-log model for postoperative day-3 neutrophil-to-lymphocyte ratio (NLR3) using heteroskedasticity-consistent type 3 (HC3) robust standard errors.

Predictor	Scale	Exp(B)	95% CI	*P*-value
Age	Per 10 years	1.011	0.844–1.212	0.903
dCA impairment proportion	Per 10 percentage points	1.022	0.816–1.280	0.848
Age × dCA impairment proportion	10 years × 10 percentage points	1.193	0.975–1.459	0.086
Baseline NLR	Per 1 unit	1.010	0.888–1.149	0.878
Operation duration	Per hour	1.214	1.041–1.414	0.013*
Female sex	Vs. male	0.858	0.537–1.371	0.521

All terms were included simultaneously. Age and dCA impairment proportion were centered at their sample means and scaled per 10 years and 10 percentage points, respectively. Male sex was the reference category. Exp(B) denotes the multiplicative change in expected NLR3. CI, confidence interval; dCA, dynamic cerebral autoregulation; HC3, heteroskedasticity-consistent type 3; NLR, neutrophil-to-lymphocyte ratio; NLR3, NLR on postoperative day 3.

*P < 0.05,

**P < 0.01.

Operation duration was associated with higher NLR3 (Exp(B) = 1.214 per hour; HC3 95% CI, 1.041–1.414; *P* = 0.013). The conditional main effects of age, dCA impairment proportion, NLR0, and sex were not statistically significant in the primary model.

### 3.3 Age-conditional associations

**FIGURE 2:**
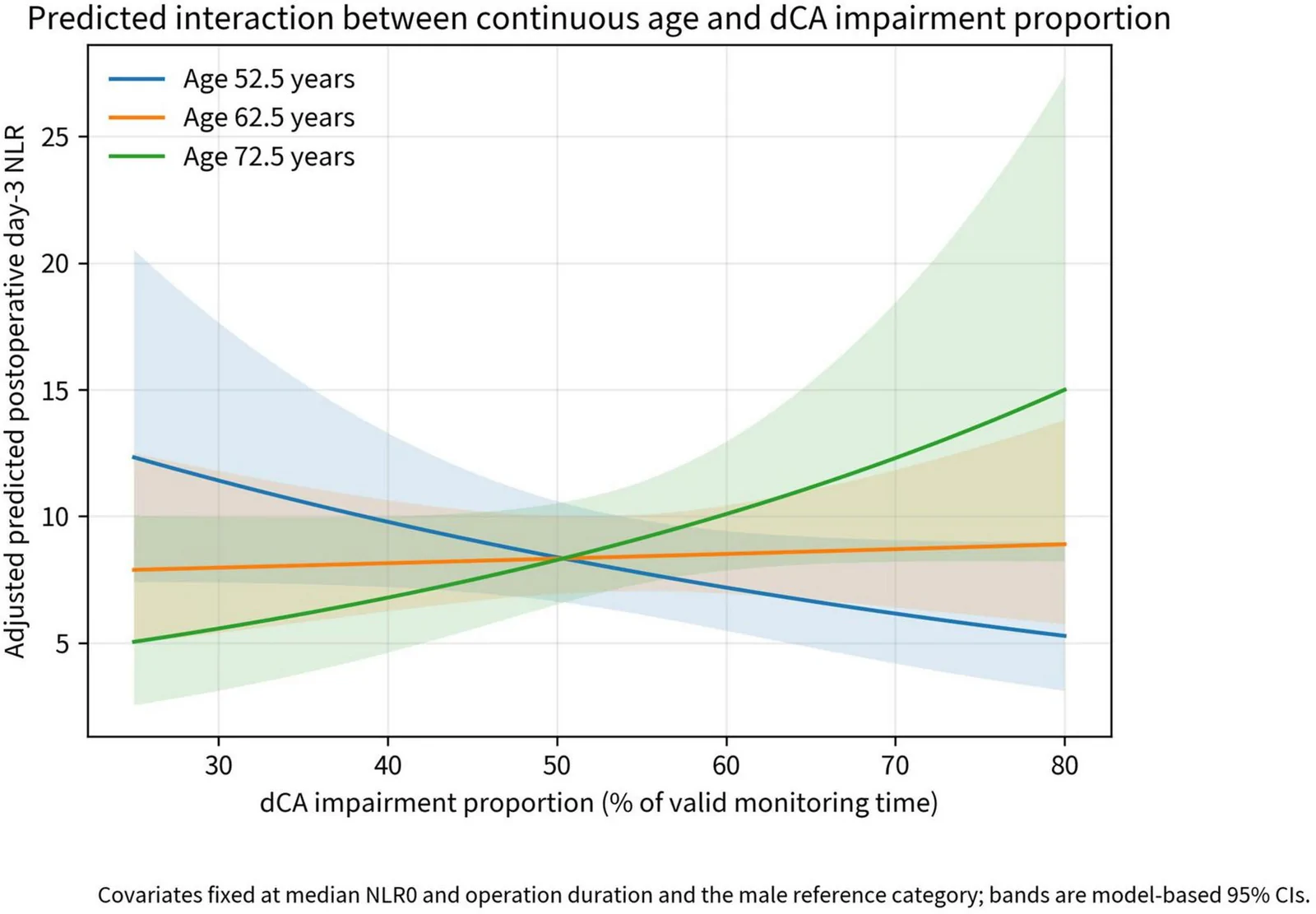
Predicted interaction between continuous age and dCA impairment proportion.

For each 10-percentage-point increase in dCA impairment proportion, the estimated multiplicative change in NLR3 was 0.857 at age 52.5 years (HC3 95% CI, 0.603–1.218; *P* = 0.390), 1.022 at age 62.5 years (95% CI, 0.816–1.280; *P* = 0.848), and 1.219 at age 72.5 years (95% CI, 0.957–1.553; *P* = 0.108) (Table 3). The confidence intervals were wide, and none of the individual age-conditional slopes reached statistical significance. Adjusted predictions across the observed dCA impairment proportion range are shown in Figure 2.

**TABLE 3:** Primary interaction estimates, bootstrap interval, and age-conditional slopes.

Analysis	Effect scale	Estimate	95% CI	*P*-value
Primary interaction: model-based SE	Exp(B)	1.193	1.051–1.354	0.006**
Primary interaction: HC3 robust SE	Exp(B)	1.193	0.975–1.459	0.086
2,000-replicate bootstrap percentile interval	B	0.176	0.051–0.370	–
Simple slope: 10 years below mean age	Exp(B)	0.857	0.603–1.218	0.390
Simple slope: mean age	Exp(B)	1.022	0.816–1.280	0.848
Simple slope: 10 years above mean age	Exp(B)	1.219	0.957–1.553	0.108

Age-conditional slopes estimate the multiplicative change in expected NLR3 per 10-percentage-point increase in dCA impairment proportion. Mean age was 62.5 years; the evaluated ages were 52.5, 62.5, and 72.5 years. The bootstrap interval is reported on the unexponentiated coefficient (B) scale; all other estimates are Exp(B). HC3 CIs are shown for conditional slopes. B, regression coefficient; CI, confidence interval; dCA, dynamic cerebral autoregulation; Exp(B), exponentiated regression coefficient; HC3, heteroskedasticity-consistent type 3; NLR3, neutrophil-to-lymphocyte ratio on postoperative day 3; SE, standard error.

*P < 0.05,

**P < 0.01.

### 3.4 Sensitivity analyses and model diagnostics

All 2,000 bootstrap models converged, and the percentile 95% interval for the interaction coefficient B was 0.051–0.370. Leave-one-out interaction estimates remained positive (B range 0.144–0.243). One observation had high leverage and a Cook’s distance of 3.72; the observation was retained, and its influence was evaluated through robust, bootstrap, and leave-one-out analyses.

**FIGURE 3:**
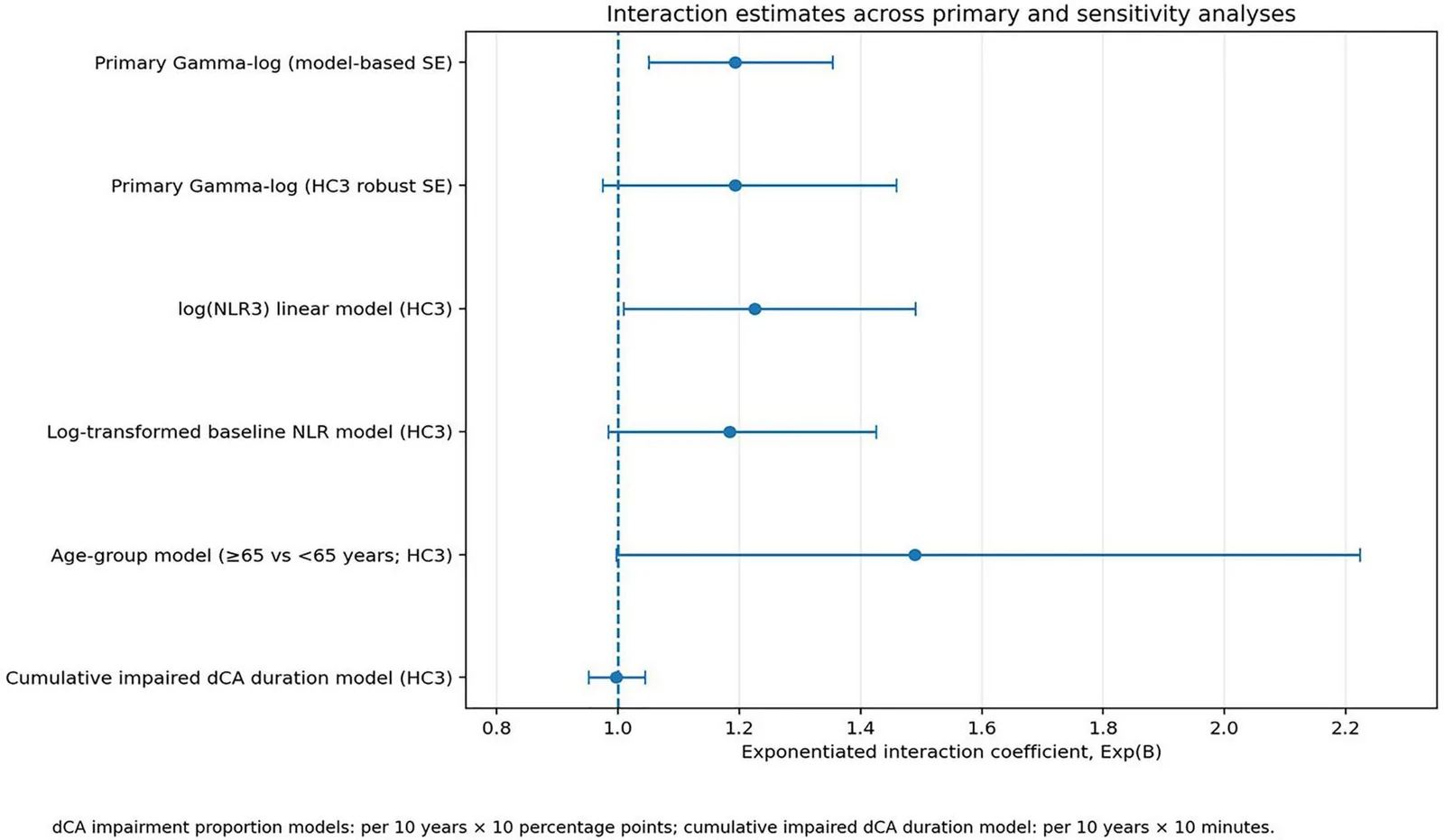
Exponentiated age × dynamic cerebral autoregulation (dCA) interaction estimates across the primary and sensitivity analyses.

The log(NLR3) linear model yielded Exp(B) = 1.226 for the interaction (HC3 95% CI, 1.009–1.490; *P* = 0.040). The Gamma model with log-transformed NLR0 yielded Exp(B) = 1.184 (HC3 95% CI, 0.984–1.426; *P* = 0.074). When age was categorized at 65 years, the age-group × dCA impairment proportion interaction was Exp(B) = 1.489 (HC3 95% CI, 0.997–2.223; *P* = 0.052). The age × cumulative impaired dCA duration interaction was not evident (Exp(B) = 0.997 per 10-year × 10-min increment; HC3 95% CI, 0.952–1.045; *P* = 0.915) (Figure 3 and Table 4).

**TABLE 4:** Sensitivity analyses for age × dynamic cerebral autoregulation (dCA) interactions.

Sensitivity analysis	Interaction	Exp(B)	95% CI	*P*-value
log(NLR3) linear model (HC3)	Age × dCA impairment proportion	1.226	1.009–1.490	0.040*
Gamma model with log-transformed baseline NLR (HC3)	Age × dCA impairment proportion	1.184	0.984–1.426	0.074
Age-group Gamma model (≥65 vs. <65 years; HC3)	Age group × dCA impairment proportion	1.489	0.997–2.223	0.052
Cumulative impaired dCA duration Gamma model (HC3)	Age × cumulative impaired dCA duration	0.997	0.952–1.045	0.915

All CIs and *P*-values are based on HC3 robust standard errors. The cumulative impaired dCA duration interaction is scaled per 10 years × 10 min; dCA impairment proportion interactions are scaled per 10 years × 10 percentage points. Exp(B) values in the log(NLR3) linear model were obtained by exponentiating the interaction coefficient. CI, confidence interval; dCA, dynamic cerebral autoregulation; Exp(B), exponentiated regression coefficient; HC3, heteroskedasticity-consistent type 3; NLR, neutrophil-to-lymphocyte ratio; NLR3, NLR on postoperative day 3.

*P < 0.05,

**P < 0.01.

Collinearity was low in the primary dCA impairment proportion model (maximum VIF, 1.20; maximum condition index, 6.85). In the cumulative impaired dCA duration model, the maximum VIF was 3.97 and the maximum condition index was 13.31, indicating greater dependence between cumulative impaired dCA duration and operation duration. Spearman correlation analyses are presented in Supplementary Table S1. All Gamma models converged; additional fit and influence measures are shown in Table 5.

**TABLE 5:** Model fit, collinearity, and influence diagnostics.

Diagnostic	Primary dCA impairment proportion model	Cumulative impaired dCA duration model
*N*	57	57
Converged	Yes	Yes
Gamma dispersion (MLE)	0.324	0.359
Residual deviance/df	0.389	0.434
Pearson χ^2^/df	0.400	0.448
Maximum VIF	1.20	3.97
Maximum condition index	6.85	13.31
Maximum Cook’s distance (primary model)	3.72 (case 55)	–
Leave-one-out interaction B range	0.144–0.243	–

The high-leverage observation was retained. Robust, bootstrap, and leave-one-out analyses were used to assess its influence. B, regression coefficient; dCA, dynamic cerebral autoregulation; df, degrees of freedom; MLE, maximum likelihood estimation; N, number of observations; VIF, variance inflation factor; χ^2^, chi-square statistic.

## 4 Discussion

### 4.1 Principal findings and statistical interpretation

The principal finding of this study was that the fitted interaction suggested that the modeled association became more positive with age between dCA impairment proportion and NLR3: as age increased, the estimated association shifted in a more positive direction. This pattern was supported by the model-based analysis, bootstrap interval, leave-one-out estimates, and the log(NLR3) sensitivity model. At the same time, the HC3 confidence interval for the primary Gamma model included the null, the age-conditional slopes were imprecise, and no interaction was detected for cumulative impaired dCA duration. Taken together, the results support an age-related pattern for dCA impairment proportion while showing that its magnitude and certainty depend on the analytic specification.

### 4.2 Biological interpretation of the age-related variation in the dCA–NLR3 association

Age was modeled continuously because the vascular, autonomic, and immune changes associated with aging develop progressively rather than beginning at a single chronological threshold. Advancing age is associated with arterial stiffening, endothelial dysfunction (Burkhart et al., 2011; Ungvari et al., 2025), altered cerebrovascular reactivity (Finger et al., 2022), reduced autoregulatory reserve (Ungvari et al., 2025), microglial priming (Dilger and Johnson, 2008; Jurgens and Johnson, 2012), immunosenescence (Mohapatra et al., 2023), and impaired autonomic regulation of inflammation (Giunta et al., 2024). In this context, the same proportion of intraoperative time with pressure-passive cerebral physiology may not represent an equivalent physiological challenge at different ages. In an older patient with reduced cerebrovascular reserve, a given proportional dCA impairment burden may occur against a lower capacity to buffer repeated arterial pressure fluctuations, whereas a younger patient may tolerate a similar monitored burden without a comparable systemic response. This interpretation remains conceptual, because cerebrovascular reserve and vascular stiffness were not measured directly in the present study.

One possible explanation for the increasingly positive dCA–NLR3 slope with age is that impaired autoregulation and the postoperative leukocyte response are parallel manifestations of a broader perioperative stress state rather than a simple causal sequence in which dCA impairment directly increases NLR. Major surgery activates sympathetic and hypothalamic–pituitary–adrenal signaling, and catecholamine and cortisol responses can promote granulocyte mobilization and redistribution of circulating lymphocytes (Giunta et al., 2024; Hamid et al., 1984; Pan et al., 2025; Silva et al., 2024; Toft et al., 1994). Age-related autonomic imbalance, endothelial dysfunction, and immune remodeling could alter the magnitude or persistence of these responses, thereby making proportional pressure passivity more closely associated with postoperative neutrophilia, lymphopenia, or both at older ages (Bowirrat, 2022; Finger et al., 2022; Giunta et al., 2024; Mohapatra et al., 2023). However, catecholamines, cortisol, cytokines, endothelial biomarkers, and autonomic indices were not measured. The present data therefore do not establish that any of these pathways mediated the observed interaction. Experimental and clinical work supports the biologic plausibility of surgery-related lymphocyte redistribution and hormone-associated granulocyte mobilization, but not a causal pathway from COx-defined impairment to leukocyte changes in this cohort.

The negative fitted slope at the younger reference age should not be interpreted as evidence that impaired autoregulation is protective. Its confidence interval was wide and compatible with both a negative and a positive association, and none of the age-conditional slopes reached statistical significance. In a small interaction model, divergence of fitted slopes may partly reflect limited information at the joint extremes of age and dCA impairment proportion, residual confounding, or sampling variability. The interaction should therefore be interpreted narrowly as a modeled change in the dCA impairment proportion–NLR3 slope across age, rather than as evidence of benefit in younger patients or harm above a specific age threshold. The borderline result obtained with the 65-years grouping further argues against an abrupt biological transition at age 65.

### 4.3 Exposure definition and the role of operation duration

The different results obtained with dCA impairment proportion and cumulative impaired dCA duration are important for interpretation. Cumulative impaired dCA duration increases with the length of the monitoring period and can therefore be correlated with operation duration even when the underlying fraction of impaired time is unchanged. In this study, the cumulative impaired dCA duration model had higher VIF and condition-index values than the dCA impairment proportion model, and its age interaction was null. dCA impairment proportion may better represent relative exposure to pressure-passive physiology during the observed period, whereas cumulative impaired dCA duration represents absolute exposure time. Neither measure is intrinsically superior; they answer related but distinct questions. Operation duration remained positively associated with NLR3 after adjustment. Longer procedures may involve greater tissue injury, physiologic disturbance, anesthetic exposure, and surgical complexity, each of which can contribute to postoperative leukocyte changes (Dobson, 2015; Liu et al., 2025). Operation duration may also act as a common determinant of both the opportunity to accumulate impaired dCA time and the magnitude of the postoperative stress response. This finding underscores the importance of separating relative dCA burden from the duration of the surgical insult.

The positive age interaction observed for impairment proportion but not for cumulative duration may indicate that age-related heterogeneity is more closely associated with the relative occurrence of pressure-passive physiology than with absolute impaired time. However, alternative explanations include the stronger dependence of cumulative duration on operation duration, differences in measurement error, and loss of information caused by threshold-based exposure construction. The present findings therefore do not demonstrate that impairment proportion is intrinsically or biologically superior. Rather, they show that the estimated interaction was sensitive to how dCA burden was defined. Future studies should prespecify both relative and absolute exposure measures and consider additional indices incorporating episode duration, frequency, laterality, and severity above the COx threshold.

### 4.4 Interpretation of NLR3 and potential biological pathways

Neutrophil-to-lymphocyte ratio on postoperative day 3 was selected because it is routinely available and reflects the balance between postoperative neutrophilia and lymphopenia. However, the same NLR value can arise from different combinations of neutrophil and lymphocyte counts, and NLR is influenced by infection, medications, malignancy, tissue injury, transfusion, and recovery (Silva et al., 2024; Zhu et al., 2023). Perioperative studies also suggest that postoperative NLR and its trajectory may contain information that is not captured by the preoperative value alone (Kim et al., 2021). Accordingly, we interpret NLR3 as a peripheral marker of the postoperative systemic response rather than as a direct measure of neuroinflammation or immune competence.

Several pathways could be explored in future work. Cerebrovascular pressure passivity may coexist with sympathetic and hypothalamic–pituitary–adrenal activation, endothelial signaling, and neuroimmune communication, while aging may alter each component of these responses. Autonomic anti-inflammatory pathways and age-related immune remodeling also provide plausible biological contexts (Borovikova et al., 2000; Giunta et al., 2024; Pan et al., 2025; Tracey, 2002). The present data do not distinguish among these pathways. Demonstrating functional immune suppression would require complementary measures such as serial absolute leukocyte counts, cytokines, lymphocyte subsets, monocyte human leukocyte antigen-DR (HLA-DR) expression, ex vivo immune responses, and infection-related outcomes (Fumeaux and Pugin, 2006).

### 4.5 Statistical robustness and effect magnitude

The interaction point estimate was stable across model-based and HC3 analyses, but the estimated precision differed. HC3 standard errors are valuable when heteroscedasticity and influential observations are concerns, especially in a small sample, and their wider interval tempers the strength of the primary inference. The positive bootstrap interval and consistently positive leave-one-out coefficients indicate that the direction was not produced by the removal of any single participant, although the high-leverage observation contributed to uncertainty.

The interaction coefficient describes how the slope relating dCA impairment proportion to NLR3 changes with age; it is not the effect of impairment at a particular age. The age-specific estimates illustrate this distinction. Even at age 72.5 years, a 10-percentage-point increase in impairment proportion corresponded to an estimated 21.9% higher expected NLR3, but the confidence interval included no association. The present data therefore provide an estimate for future sample-size planning rather than a threshold for individual risk prediction.

### 4.6 Clinical and research implications

These findings encourage closer study of age when linking intraoperative cerebrovascular physiology with postoperative systemic responses. They do not indicate that impaired autoregulation is beneficial at any age. In clinical practice, cerebral perfusion and blood pressure should continue to be managed according to the patient’s condition and established hemodynamic principles. The value of the present observation lies in identifying a testable age-related association and in showing that dCA impairment proportion and cumulative impaired dCA duration may convey different information.

A prospective multicenter study should include all consecutively monitored patients, including those without COx-defined impairment; retain complete device and signal-processing metadata; prespecify continuous age-interaction analyses; and collect serial NLR and its component cell counts. Additional cytokine, cellular, autonomic, infection, delirium, cognitive, and recovery outcomes would allow the statistical interaction to be related to specific biological and clinical pathways. Larger samples could also evaluate nonlinear age effects and identify whether the association differs across surgical types or disease states.

### 4.7 Strengths and limitations

Strengths of the study include continuous intraoperative cerebral physiology, use of dCA impairment proportion alongside cumulative impaired dCA duration, adjustment for baseline NLR and operation duration, clinically interpretable scaling of the interaction, and multiple assessments of model stability. Reporting both model-based and robust uncertainty estimates, together with bootstrap, leave-one-out, collinearity, and influence analyses, makes the limits of the small-sample estimate explicit.

Several limitations should be considered. First, the single-center cohort was small, and the interaction and age-conditional slopes remain imprecise. Second, this nested cohort included monitored patients with recorded COx-defined impairment; the number and characteristics of otherwise eligible patients who were not monitored were not available, which may introduce selection bias. Third, NLR was measured at a single postoperative time point, and the study did not include serial NLR measurements, changes in NLR, cytokines, cell phenotypes, or functional immune outcomes. Fourth, residual confounding may remain from cancer type and stage, neoadjuvant treatment, corticosteroids, infection, transfusion, postoperative complications, intensive care unit admission, analgesia, and other perioperative factors.

Fifth, two high NLR3 values and one high-leverage observation affected model diagnostics, although sensitivity analyses preserved the direction of the dCA impairment proportion interaction. Sixth, the interaction was not supported by every analytic specification, particularly the HC3 primary *P*-value and the cumulative impaired dCA duration model. Seventh, device-specific sampling, moving-window, synchronization, and artifact-rejection parameters were not retained in the analytic export available for this nested study, limiting full reproduction of the COx signal-processing pipeline. Finally, the study was not designed to estimate treatment effects or to link the interaction directly with neurologic or infectious outcomes.

## 5 Conclusion

Among adults undergoing major abdominal surgery, the modeled association between dCA impairment proportion and NLR3 became more positive with increasing age. The interaction was consistent in several stability analyses but was less precise with HC3 inference and was not reproduced when cumulative impaired dCA duration was used. The findings define an age-related signal for further investigation and support prospective studies that combine standardized dCA monitoring with serial cellular, inflammatory, and clinical outcomes.

## Data Availability

The datasets presented in this article are not readily available because data cannot be shared currently, as this nested study belongs to an ongoing parent cohort study. Raw data release is restricted to avoid premature data disclosure; dataset will be available upon formal completion of the main project and can be requested from the corresponding author afterwards. Requests to access the datasets should be directed to YL, Liuyt@Lzu.edu.cn.

## Footnotes

1
https://icmplus.neurosurg.cam.ac.uk/
