# Supplementary material for: Age-related variation in the association between intraoperative dynamic cerebral autoregulation impairment proportion and postoperative neutrophil-to-lymphocyte ratio: an exploratory observational study
Source: Front Aging Neurosci. 2026 Sep 1;18:1898034. doi: 10.3389/fnagi.2026.1898034 (PMC13574702; doi:10.3389/fnagi.2026.1898034)
Supplement: Supplementary file 1 [file Data_Sheet_1.docx]

Figure S1. Box plot of NLR3.

Figure S2. Histogram of NLR3.

Figure S3. Histogram-age group distribution of NLR3.

Figure S4. Q–Q plot of NLR3.

| Variable Pair | Correlation coefficient, r | *P* value |
| --- | --- | --- |
| Operation duration × Cumulative impaired duration (at least one hemisphere) | 0.705** | < 0.001 |
| Valid monitoring time × Cumulative impaired duration (at least one hemisphere) | 0.800** | < 0.001 |
| Operation duration × dCA impairment proportion | −0.047 | 0.727 |
| Valid monitoring time × dCA impairment proportion | −0.053 | 0.695 |

Table S1 Spearman correlations for dCA impairment measures.

Note: dCA, dynamic cerebral autoregulation. Correlation coefficients and *P* values were derived from Spearman rank correlation analysis. ** *P* < 0.001.
